# Pazopanib in advanced soft tissue sarcomas

**DOI:** 10.1038/s41392-019-0049-6

**Published:** 2019-05-17

**Authors:** Alex T. J. Lee, Robin L. Jones, Paul H. Huang

**Affiliations:** 1 Division of Molecular Pathology, The Institute of Cancer Research, London, UK; 2 Sarcoma Unit, The Royal Marsden NHS Foundation Trust, London, UK; 3 Division of Clinical Studies, Institute of Cancer Research, London, UK

**Keywords:** Sarcoma, Sarcoma, Cancer therapy

## Abstract

Pazopanib is the first and only tyrosine kinase inhibitor currently approved for the treatment of multiple histological subtypes of soft tissue sarcoma (STS). Initially developed as a small molecule inhibitor of vascular endothelial growth factor receptors, preclinical work indicates that pazopanib exerts an anticancer effect through the inhibition of both angiogenic and oncogenic signaling pathways. Following the establishment of optimal dosing and safety profiles in early phase studies and approval for the treatment of advanced renal cell carcinoma, pazopanib was investigated in STS. A landmark phase III randomized study demonstrated improved progression-free survival with pazopanib compared to that with placebo in pretreated patients with STS of various subtypes. The efficacy of pazopanib in specific STS subtypes has been further described in real-world-based case series in both mixed and subtype-specific STS cohorts. At present, there are no clinically validated predictive biomarkers for use in selecting patients with advanced STS for pazopanib therapy, limiting the clinical effectiveness and cost-effectiveness of the drug. In this review, we summarize the preclinical and clinical data for pazopanib, outline the evidence base for its effect in STS and explore reported studies that have investigated putative biomarkers.

## Introduction

Pazopanib is an oral multitarget tyrosine kinase inhibitor (TKI) with a clinical antitumor effect that is thought to be exerted through its selective inhibition of vascular endothelial growth factor receptor (VEGFR)-mediated angiogenesis, as well as its direct blockade of growth-promoting receptor tyrosine kinases (RTKs), including platelet-derived growth factor receptors (PDGFRs), fibroblast growth factor receptors (FGFRs), and KIT^1–5^. After receiving marketing authorization for the treatment of metastatic renal cell carcinoma (mRCC), pazopanib became the first (and currently only) TKI licensed for the treatment of multiple subtypes of advanced soft tissue sarcoma (STS). This approval was based on the results of a double-blind, placebo-controlled randomized phase III trial that demonstrated significant prolongation of progression-free survival (PFS) in patients with pretreated advanced STS who received pazopanib^6^. However, despite this evidence of an antitumor effect, no significant difference in overall survival (OS) was observed between pazopanib and placebo-treated patients. The failure of PFS gain to translate to OS benefit has adversely influenced the cost assessment of pazopanib for this indication, leading to funding limitations in certain health economies worldwide^7–9^. There is currently a poor understanding of the clinical mechanisms of pazopanib response and resistance and an unmet need for predictive biomarkers that are able to prospectively select the subgroup of STS patients most likely to benefit from pazopanib, thus improving the clinical efficiency of the drug. In this review, we summarize the preclinical and early clinical development of pazopanib, explore the evidence for efficacy in STS and outline reported data resulting from efforts to identify biomarkers for pazopanib response.

## Preclinical development of pazopanib

**Fig. 1:**
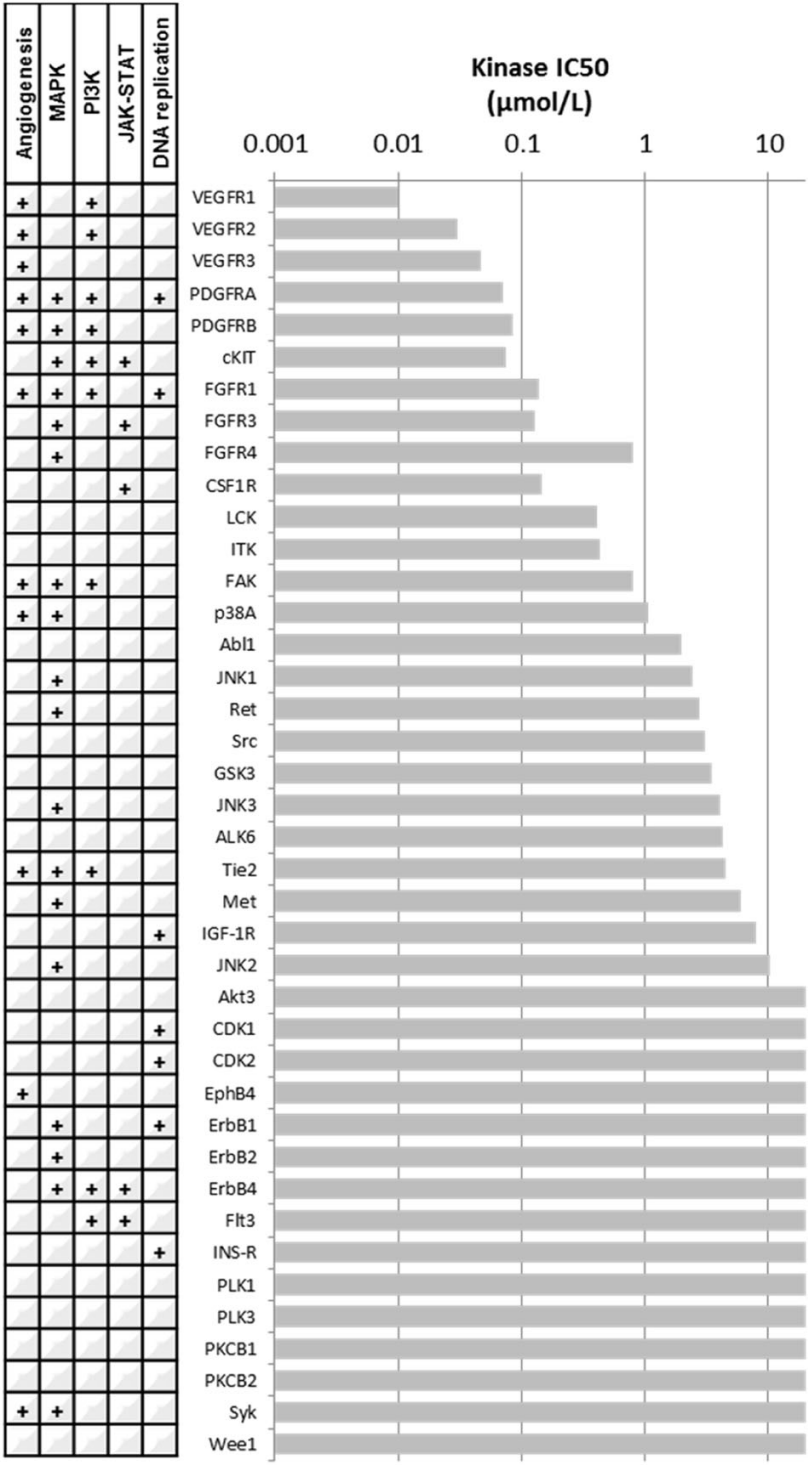
Kinase inhibitory profile of pazopanib. The bar graph indicates the kinase inhibitory concentration (IC50—drug concentration at which 50% of the target enzymatic activity is inhibited in a cell-free kinase assay) of pazopanib, as reported by Kumar et al.^3^ Involvement of kinases in canonical oncogenic processes/pathways is indicated in the table on the left (GO.0001525—angiogenesis; GO.0043410—positive regulation of MAPK cascade; GO.0014068—positive regulation of phosphatidylinositol 3-kinase signaling; GO:0046427—positive regulation of the JAK-STAT cascade; GO.0045740—positive regulation of DNA replication)

Pazopanib was identified through the chemical screening of compounds for inhibition of VEGFR2, a key mediator of tumor angiogenesis^10^. After identification of an initial lead compound as an inhibitor of VEGFR2 by using a direct kinase activity assay, chemical optimization was undertaken to increase the inhibitory potency and improve the pharmacokinetic (PK) profile in mouse models. Optimized molecules were also shown to have inhibitory potency against VEGFR1 and VEGFR3, as well as other closely-related RTKs, including PDGFRB, KIT, FGFR1, and colony-stimulating factor 1 receptor (CSF1R). This study and further reports on the kinase inhibitory profile of pazopanib are summarized in Fig. 1^1–3^. Pharmacodynamic (PD) studies of the biological activity of the drug were performed in a range of in vitro and in vivo assays^3^. Treatment of cultured human umbilical vein endothelial cells (HUVECs) with pazopanib resulted in potent inhibition of VEGF-mediated phosphorylation of VEGFR2, with associated inhibition of HUVEC proliferation. In vitro inhibition of ligand-mediated phosphorylation of KIT and PDGFRB by pazopanib was also shown in human lung cancer and foreskin fibroblast cells, respectively, although the drug had no effect on proliferation in an unspecified panel of tumor cells. Inhibition of angiogenesis was demonstrated in a mouse model of ocular angiogenesis and subcutaneous implantation of a ligand-containing Matrigel plug. Administration of oral pazopanib to immunocompromised mice was associated with dose-dependent inhibition of growth of established xenografts of human colorectal, melanoma, prostate, breast, kidney, and breast cancer cell lines. A steady-state plasma concentration was shown to provide optimal in vivo inhibition of VEGFR2 phosphorylation, angiogenesis, and xenograft growth, with a strong correlation between inhibition of VEGFR2 kinase activity and antitumor effect in xenografts.

Further preclinical evidence has indicated that the antitumor effect of pazopanib may not solely be mediated by inhibition of angiogenesis but may also be mediated through a direct effect on tumor cells. A study investigating the effect of pazopanib in human multiple myeloma models found that, in addition to the inhibition of endothelial cell proliferation and in vivo tumor angiogenesis, the drug also had direct antiproliferative and proapoptotic effects on tumor cells and xenografts^5^. This effect on myeloma cells correlated with downregulation of several cancer-related genes involved in pathways including cytokine and chemokine signaling, cell cycle, and insulin receptor pathways, as well as upregulation of proapoptotic genes. Pazopanib has been shown across a series of studies to act as a pan-RAF inhibitor and to exert an anticancer effect through inhibition of MAPK pathway signaling in cancer cells in the absence of a demonstrable antiangiogenic effect^11^. In in vivo and xenograft models of a brain-tropic Her2-positive, BRAF-mutated breast cancer cell line, pazopanib prevented the growth of brain metastasis in association with reduced MAPK pathway activation but no change in markers of angiogenesis. A later study in the same breast cancer xenograft model showed that the inhibition of brain metastasis growth by pazopanib was accompanied by a reduction in the number of PDGFRB-expressing, metastasis-associated astrocytes, suggesting a possible role for pazopanib-mediated therapeutic modulation of the tumor microenvironment (TME)^12^. In follow-on studies, a panel of breast cancer and melanoma cell lines with varying *BRAF* mutational status were used in orthotopic xenograft models that were then treated with pazopanib^13^. Here, xenografts with either wildtype or exon-11-mutated *BRAF* showed significant sensitivity to pazopanib and a corresponding reduction of MAPK pathway activation in tumor cells and reduced angiogenesis.

Collectively, these preclinical data demonstrate that pazopanib is a potent inhibitor of several key kinases involved in angiogenic and oncogenic pathways, with an antitumor effect that is mediated by both antiangiogenic and direct anticancer cell activity.

## Early phase clinical development of pazopanib

Based on these preclinical findings of antitumour effects and proposed optimal dosing, a phase I trial of pazopanib was performed, with 43 patients enrolled in an initial dose-escalation phase and a further 20 patients in a subsequent dose-expansion phase^14^. PK assessment identified that steady-state exposure was achieved at doses of 800 mg or more as a once daily oral dose. In line with toxicities observed with other antiangiogenic TKIs, hypertension was the most common adverse event (grade 3 in 25%), followed by diarrhea, hair depigmentation, nausea, anorexia, and fatigue. Proteinuria was the most common laboratory abnormality (any grade seen in 52% of patients), followed by a range of cytopenias and blood biochemistry disturbances, which were grade 1 and 2 in the large majority of affected patients. As no maximally tolerated dose was identified, an oral dose of 800 mg once daily was selected for further studies because doses > 800 mg did not increase drug exposure.

PD analyses in this phase I study demonstrated that plasma VEGF concentrations increased by more than three-fold in ~50% of treated patients following drug initiation. In a subset of patients who underwent dynamic contrast-enhanced magnetic resonance imaging (DCE-MRI), 7/12 (58%) patients were seen to have a >50% reduction in tumor blood flow at Day 8 of treatment, and 10/11 (91%) at Day 22. The incidence of hypertension was associated with higher trough drug levels on Day 22 of therapy, suggesting that hypertension may act as a PD marker of pazopanib activity.

Assessment of preliminary clinical activity in this study recorded a partial response by RECIST criteria in three patients (two with mRCC and one with pancreatic adenocarcinoma), while stable disease of at least 6 months duration was observed in 14 patients—of note, among these were two patients with chondrosarcoma, one with leiomyosarcoma (LMS), and one with a gastrointestinal stromal tumor (GIST). A further phase I trial to assess PK and PD in 53 patients aged 2–22 years was also undertaken and demonstrated a similar toxicity profile to that seen in adult patients, with one patient with occult brain metastasis experiencing intracranial bleeding^15^. All patients who underwent DCE-MRI evaluation of tumor vascular dynamics demonstrated decreases in tumor blood flow and permeability, while two objective partial responses (one with desmoplastic small round cell tumor (DSRCT)) and stable disease of >6 months in eight patients (seven with sarcomas) were observed.

Based on these phase I data, pazopanib was deemed to be a safe and generally well-tolerated drug with an optimal oral dose of 800 mg once daily. Early evidence of clinical efficacy prompted further development in mRCC, a cancer with a well-described central role of angiogenesis in tumor development. Subsequent randomized phase III trials in mRCC demonstrated superior PFS with pazopanib vs. placebo in pretreated patients and noninferior disease control and survival. In addition, pazopanib showed favorable quality-of-life outcomes compared to sunitinib, another antiangiogenic TKI already approved for first line treatment^16,17^. These studies established pazopanib as a standard of care in mRCC while also providing further data on drug toxicity, confirming severe hypertension as a commonly encountered adverse effect, as well as significant neutropenia and/or liver enzyme derangement in ~10% of patients.

## Clinical development of pazopanib for advanced STS

As a result of the evidence of durable disease stabilization seen in 4/9 patients with sarcomas treated with pazopanib in the initial phase I trial, further development of the drug in these diseases was pursued.

### EORTC noncomparative phase II trial

A noncomparative phase II trial of pazopanib was undertaken by the European Organization for Research and Treatment of Cancer (EORTC) Soft Tissue and Bone Sarcoma Group (STBSG) in patients with advanced intermediate or high-grade STS with confirmed disease progression, who were either ineligible for cytotoxic chemotherapy or who had received fewer than three prior cytotoxic agents for advanced disease^18^. Although this study was nonrandomized, patients were prospectively stratified into one of four histologically defined strata: LMS, liposarcoma (LPS), synovial sarcoma (SS) and a heterogeneous ‘other’ stratum. The primary efficacy endpoint was the progression-free rate at 12 weeks of treatment (12wPFR), with predefined rates of 40% and 20% determined as thresholds reflecting drug activity or inactivity^19^. For each stratum, an initial enrollment of 17 evaluable patients was to be treated at 800 mg once daily until disease progression, unacceptable toxicity, or withdrawal of consent. If 4/17 patients experienced nonprogression at 12 weeks, that stratum was expanded to a total accrual of 37 patients, and if 11 or more out of 37 patients were progression-free, this would indicate that further investigation of pazopanib in that stratum would be warranted.

A total of 142 patients were treated within the study. In the LPS arm, only 3 of the first 17 patients showed nonprogression at 12 weeks, and thus, recruitment to this cohort was terminated due to lack of activity. The three other arms continued to complete the second stage of recruitment. Included among the 44 patients recruited into the ‘other subtypes’ stratum were small numbers of malignant peripheral nerve sheath tumors (MPNSTs), vascular sarcomas, fibrohistiocytic or fibroblastic sarcomas, solitary fibrous tumors, rhabdomyosarcoma and GIST, as well as 19 tumors of unspecified differentiation. Four out of 142 patients were excluded from the efficacy analysis due to lack of measurable disease, change of diagnosis to GIST on central histopathology review or lack of objective disease progression prior to commencement of pazopanib. A further two patients were not evaluable due to resection of the target lesion or withdrawal due to coronary heart disease, resulting in a total of 136 patients evaluable for efficacy.

Although accrual of the LPS arm was stopped after the first stage, two patients who had initially been categorized into one of the other three strata were found on central histopathology review to have LPS. As both of these patients met the 12-week progression-free endpoint, the overall 12wPFR in the LPS arm was 26% (5/19 patients). In the LMS, SS and ‘other’ strata, 12wPFR was 44% (18/41 patients), 49% (18/37), and 39% (16/41), respectively. An objective partial response was seen in 9/136 patients (6.6%): 5 SS, 3 ‘other’, and 1 LMS. Of these patients, 4 (3 other, 1 SS) experienced disease progression between 253 and 503 days after treatment initiation, while the other 5 (1 LMS, 4 SS) were still progression free at 415–812 days. The median PFS was 80, 91, 161, and 91 days, and the median OS was 197, 354, 310, and 299 days in the LPS, LMS, SS, and ‘other’ strata, respectively. These PFS and OS rates compared favorably to the historic controls for LMS, SS, and other subtype cohorts^19^.

### PALETTE phase III randomized control trial (RCT)

Building on the results of the EORTC phase II study, the STBSG, in collaboration with GlaxoSmithKline (the manufacturer of pazopanib), undertook the Pazopanib explored in SofT-Tissue Sarcoma (PALLETTE) study, a double-blinded, placebo-controlled phase III RCT^6^. In this international, multicenter trial, adult patients with progressing advanced STS were randomized on a 2:1 basis to receive pazopanib 800 mg once daily or placebo until disease progression, unacceptable toxicity or patient withdrawal. On-trial crossover to pazopanib was not permitted for patients who progressed on placebo. Eligibility criteria stipulated that patients must have received between 1 and 4 lines of previous systemic therapy, including an anthracycline, for advanced disease. Most common histological subtypes of STS were eligible for enrollment—notably, based on the earlier phase II evidence of limited activity, LPS was not included in this phase III trial. PFS was the primary endpoint, with a sample size designed to provide 95% power to detect a 15% difference in the progression-free rate at 6 months, corresponding to a hazard ratio of 0.63. This sample size would also provide 90% power to detect HR 0.67 for OS, which was included in secondary endpoints along with toxicity and quality-of-life measures.

Between October 2008 and February 2010, 369 patients were randomized, with 246 allocated to pazopanib and 123 allocated to placebo. In the analysis of the intention-to-treat cohort, after a median follow-up of 25 months, a clinically significant 3-month improvement in median PFS was seen with pazopanib (4.6 vs. 1.6 months; HR 0.31; 95% CI 0.24–0.40; *p* < 0.0001). There was no significant difference in OS (median OS 12.5 vs. 10.7 months; HR 0.86; 95% CI 0.67–1.11, *p* = 0.2514). Posttrial systemic treatment was received by 49% and 63% of patients in the pazopanib and placebo arms, respectively. Respective best objective responses as determined by an external review in the pazopanib and placebo arms were partial response in 6% and 0%, stable disease in 67% and 38%, and disease progression/death in 24% and 62% of patients, respectively. Safety and toxicity data were broadly in keeping with earlier pazopanib studies, with fatigue, hypertension, diarrhea, and anorexia among infrequently experienced grade 3–4 toxicities. A small excess in decreased left ventricular ejection fraction, thromboembolic events, and pneumothorax was observed in the pazopanib arm. One of eight on-treatment deaths in the pazopanib arm—a patient who died of multiorgan failure—was possibly related to the study drug. Subsequently, an exploratory health-related quality-of-life analysis reported that the scores for general health status did not significantly differ between pazopanib and placebo-treated patients, while specific toxicity-related measures related to diarrhea, anorexia, nausea, fatigue, and role functioning favored the placebo^20^.

A subsequent post hoc analysis of the Japanese subcohort from the PALETTE study (*n* = 47) demonstrated similar levels of PFS and OS benefit to the overall study population^21^. There was no overall difference in the type or severity of pazopanib-related toxicity within the Japanese subcohort and overall study population, although a higher rate of dose reduction and lower average daily dose was experienced by Japanese patients. The lower pazopanib exposure but equivalent efficacy seen in the Japanese cohort potentially suggests contrasting PK profiles between different ethnic groups. However, given that pazopanib dose reduction within these trials was at investigator discretion, it is possible that the more frequent dose reductions reflect regional variations in practice. A similar comparison of TKI safety in patients with mRCC treated in an RCT with pazopanib or sunitinib reported similar levels of drug exposure but a distinct pattern and severity of adverse events in Asian versus non-Asian subgroups^22^. Meanwhile, a phase I study of pazopanib in a Japanese population reported a similar PK profile to the initial phase I^23^. Although these data do not provide a consistent picture of ethnic and geographical variation in the PK and PD profile of pazopanib, recognized differences in the metabolism of many cancer and noncancer drugs between different ethnic groups indicate the potential for ethnic background to contribute to interindividual variation in pazopanib exposure and toxicity^24,25^.

A further post hoc subgroup analysis of data from the PALETTE trial, in combination with data from the preceding phase II, focused on patients with uterine sarcomas treated with pazopanib (*n* = 44, 88.6% LMS, 84.1% high grade)^26^. Compared to patients with uterine sarcomas who received placebo within the PALETTE study, patients randomized to pazopanib had a significantly longer median PFS (3.0 vs. 0.8 m, *p* < 0.001) and median OS (17.5 vs. 7.9 m, *p* = 0.038).

The results from the PALETTE study were taken to reflect a clinically meaningful level of activity and benefit from pazopanib in the eligible population, leading to the drug receiving a license for use in pretreated advanced STS of non-LPS subtypes in the US and Europe in 2012. The subgroup analyses indicated that the efficacy and safety profile of pazopanib is similar in Japanese and non-Japanese populations and that the efficacy in uterine sarcomas is broadly equivalent to that in nonuterine sarcomas.

### Other clinical evidence of pazopanib effect in STSs

Several other prospective and retrospective series provide additional evidence for the efficacy of pazopanib, both in unselected cohorts of mixed histological subtype, as well as diagnosis-specific series with a variable focus on epithelioid sarcoma, SFT, DSRCT, chondrosarcomas, and vascular sarcomas. In addition, data have been reported for 211 patients of mixed STS subtypes treated with pazopanib in an international expanded access program that was conducted following the PALETTE trial^27^. The efficacy data from these studies are summarized in Table 1 and broadly conform to those from the PALETTE study, with infrequent objective responses seen and median PFS and OS values following the start of pazopanib of ~3–5 and ~10–14 months, respectively. Of note is a prospective single arm phase II study that provides further information on the efficacy of pazopanib in LPS^28^. Opening following the reporting of the results of the PALETTE study and noting the results of the antecedent phase II where efficacy of pazopanib in the final, centrally confirmed LPS cut-off was in fact above the predetermined futility cut-off. This multicenter US study treated 41 patients with intermediate or high-grade LPS with pazopanib and reported a 12wPFR of 60%. The median PFS and OS (4.4 and 12.6 months, respectively) were consistent with efficacy data for other STS subtypes from the PALETTE study. However, particularly given that the EORTC pazopanib phase II study did not distinguish between the clinical and biological diversity encompassed within the principle LPS subtypes^29^, the precise role of pazopanib and other antiangiogenic agents in LPSs remains to be defined.

**Table 1:** Overview of the reported efficacy data from prospective and retrospective clinical trials of pazopanib monotherapy in advanced soft tissue sarcoma

Study	Prospective							Retrospective									
	Sleifjer (2009)^18^	Van der Graaf (2012)^6^	Frezza (2014)^72^	Benson (2016)^26^	Samuels (2017)^28^	Martin-Broto (2019)^45^	Maruzzo (2015)^43^	Stacchiotti (2018)^73^	Frezza (2018)^74^	Gelderblom (2017)^27^	Jones (2017)^75^	Kollar (2016)^76^	Menegaz (2017)^77^	Nakamura (2016)^78^	Nakano (2015)^79^	Stacchiotti (2014)^44^	Yoo (2015)^80^
Design	Non-comparative phase II	Double blind, placebo-controlled, phase III	Subgroups from phII and III trials + EAP data	Subgroups from phII and III trials	Single arm, multicentre US phase II	Single arm, multicentre European phase II	Single centre case series	Int’l multicentre case series	Int’l multicentre case series	Intl’ multicentre case series based on EAP	Int’l case series	European multicentre case series, inc phase II/III	UK centre case series	Japanese multicentre case series	Japanese centre case series	Italian multicentre series	Korean centre series
*N*	142	369	9	44	41	36	13	30	18	211	8	52	29	156	47	6	43
Subtypes	LMS, SS, LPS, ‘Other’	Mixed (LPS excluded	DSRCT	Uterine sarcoma (89 % LMS)	LPS (int/high grade)	SFT	SFT	ASPS	Epithelioid sarcoma	Mixed	Chondro-sarcoma	Vascular sarcomas	DSRCT	Mixed	Mixed	SFT	Mixed
Eligibility	<3 prior lines	1–3 prev lines	≥2nd line	≥2nd line	Any line	Any line	1st line	Any line	Any line	1–3 prev lines	Any line	Any line	Any line	Any line	Any line	Any line	≥2nd line
Best response: CR or PR	6%	6%	22%	11%	2%	6%	8%	27%	0%	7%	0%	23%	7%	8%	11%	0%	16%
SD	NR	67%	56%	57%	42%	60%	62%	57%	50%	18%	75%	21%	55%	47%	NR	50%	42%
PrD	NR	24%	22%	32%	66%	34%	15%	13%	50%	41%	25%	50%	38%	24%	NR	50%	37%
12wPFR	LMS:44% SS:49% LPS:26% Other:39%	60%^a^	67%	50%	68%	NR	62%	59%	50%	50%	75%	AS:45%^a^ EHE:60%^a^	62%	60%^a^	60%^a^	50%^a^	NR
Median PFS (months)	LMS:3.0 SS:5.4 LPS:2.7 Other:3.0	4.6 (vs. 1.6 in placebo arm)	9.2	3	4.4	5.6	4.7	13.6	3	3	NA	AS: 3.0 EHE: 26.3	5.6	3.6	4.3	3	5
Median OS (months)	LMS:11.8 SS:10.3 LPS:6.6 Other:10.0	12.5 (vs. 10.7 in placebo arm)	15.4	17.5	12.6	Not reached	13.3	Not reached	14	11.1	NR	AS: 9.9 EHE 26.3	15.7	11.2	9.6	NR	8.2
Comments	Favorable PFS and OS vs. historical control in LMS, SS and Other subgroups -	Drug licensed in pre-treated non-adipocytic STS based on evidence of PFS benefit		In PALETTE trial, significantly longer PFS and OS with pazopanib vs. placebo	LPS subtypes: 66% DDLPS, 29% MLPS, 5% PleoLPS	Response by Choi criteria: 51% PR 26% SD 23% PrD		Median follow-up 19 months	Authors conclude limited activity in ES		5/8 Convent-ional CS 1/8 ESMC 1/8 MC 1/8 clear cell	Equivalent ORR in cutaneous vs. non-cutaneous or 1° vs. 2° AS		NB 33/156 (21%) LPS	Trend toward better PFS in PALETTE eligible subtypes (HR 0.56, 95% CI 0.25–1.23, *p* = 0.15)		

*CR* complete response, *PR* partial response, *SD* stable disease, *PrD* progressive disease, *12wPFR* 12 week progression-free rate, *PFS* progression-free survival, *OS* overall survival, *LMS* leiomyosarcoma, *SS* synovial sarcoma, *LPS* liposarcoma, *DSRCT* desmoplastic small round cell tumor, *SFT* solitary fibrous tumor, *AS* angiosarcoma, *EHE* epithelioid hemangioendothelioma, *DDLPS* dedifferentiated liposarcoma, *MLPS* myxoid liposarcoma, *PleoLPS* pleomorphic liposarcoma, *ES* epithelioid sarcoma, *CS* chondrosarcoma, *ESMC* extraskeletal myxoid chondrosarcoma, *MC* mesenchymal chondrosarcoma, *ASPS* alveolar soft part sarcoma

^a^Approximate

In an attempt to improve the generally modest activity of pazopanib in unselected advanced STS, a number of reported and ongoing trials have investigated the combination of the drug with various cytotoxic chemotherapy regimens or targeted agents, such as histone deacetylase inhibitors^30–33^. While several of these combinations have been associated with unacceptable toxicity and/or insufficient activity in phase I studies, others have progressed to examining efficacy in STS-enriched cohorts. Due to the lack of convincing evidence of incremental activity of such combinations, further detail of these studies is not explored in this review.

## Evidence of biomarkers of pazopanib effect

The effect of pazopanib in STS is evidenced by the improvement in PFS without associated deterioration in quality of life compared to placebo in a randomized phase III study, as well as reports of postapproval experience in ‘real world’ settings. However, the benefit of pazopanib treatment to individual patients is highly variable and, in the context of infrequent objective tumor responses, often difficult to confirm. Furthermore, despite the RCT evidence of an antitumor effect in an otherwise poor-prognosis patient group with limited treatment options, no significant difference in OS was seen between pazopanib-treated and placebo-treated patients. It has not been established why a significant PFS gain did not translate into an OS benefit; proposed explanations have included a lack of statistical power, the confounding effect of subsequent posttrial treatments or a potential ‘rebound’ effect, wherein initial treatment with pazopanib may select for or induce a more aggressive subsequent phenotype or, alternatively, where accelerated progression is encountered after the cessation of treatment^34–36^. The absence of a demonstrated OS benefit in an unselected STS population has adversely affected cost-effectiveness appraisals of pazopanib, resulting in limited access to the drug in several health economies^7–9^. The ability to prospectively identify patients with advanced STS who are most likely to benefit from pazopanib would aid clinical decision making, increase the clinical and cost-effectiveness of the drug and improve patient experience and survival outcomes. However, as summarized below and in Table 2, no routinely recorded clinicopathological parameters or experimental assays have yet been shown to be consistently capable of discriminating between patients with a higher or lower chance of benefiting from pazopanib.

**Table 2:** Candidate biomarkers investigated for association with the pazopanib effect in advanced STS

Candidate biomarker	Reported association
*Baseline clinico-pathological characteristics*	
Histological subtype	•Lower 12wPFR in LPS compared to LMS, SS and heterogeneous ‘other’ subtypes^18^
	•No enrichment for particular subtype in long-term responders and/or survivors^38^
Performance status 0 Low histological grade	•Favorable PFS and OS^6,38^
*Radiological markers*	•Possible superiority of Choi criteria and/or FDG-PET over RECIST 1.1 in categorizing stable disease^47,48^
*Pharmacodynamic markers*	
Hypertension	•No association of hypertension with improved PFS or OS in post-hoc analysis of aggregated phase II/III data^52^
Other on-target toxic effects	•No association of drug-induced proteinuria, hypothyroidism or cardiotoxicity with improved PFS or OS in post-hoc analysis of aggregated phase II/III data^54^
Concomitant gastric acid suppression (GAS)	•Significantly inferior PFS (HR1.49, 95% CI 1.11–1.99, *p* = 0.008) and OS (HR 1.81, 95% CI 1.31–2.49, *p* < 0.001) among pazopanib-treated patients who received concomitant GAS^56^
*Baseline biological markers*	
Circulating angiogenic factors	•Association with worse PFS in single study—requires validation^61^
Circulating neutrophil-to-lymphocyte ratio	•Raised ratio acts asf a poor prognostic marker but not predictive for pazopanib effect^63,64^
Tumor TP53 mutation	•Association between NGS-detected *TP53* mutation and improved PFS with pazopanib—single small retrospective study^65^

### Clinicopathological parameters

Preplanned and post hoc analyses of the EORTC phase II and III trials of pazopanib have been performed in an attempt to identify patient or tumor characteristics that enrich for drug benefit. As discussed above, LPS was identified as a potentially insensitive histological subtype within the phase II study, but subsequent analysis and other studies call into question whether there are subsets of patients with LPS who may respond to treatment^18^. Within the PALETTE study cohort, multivariate analysis identified good performance status and lower histological grade as factors associated with improved outcome, both of which are well-established prognostic factors in STS regardless of pazopanib exposure^6^. Predictive analysis did not detect a significant interaction between histological subtype and pazopanib benefit, with improved PFS with pazopanib vs. placebo seen in all three histological subgroups (LMS, SS, other). Other baseline characteristics, including gender, ethnicity, and extent of prior treatment, were not found to be predictive for OS^37^.

Patients who received pazopanib within the EORTC phase II and phase III trials and who met PALETTE-eligibility criteria (i.e., non-adipocytic histology, measurable disease, adequate organ function, etc.) (*n* = 344) were included in a retrospective analysis that sought to identify baseline factors associated with a good outcome following treatment^38^. Here, the authors defined PFS ≥ 6 months and OS ≥ 18 months as long-term response and survival, respectively, with 36% of patients showing long-term response, 34% showing long-term survival, and 22% showing both long-term response and survival. Descriptive and multivariate analyses again identified tumor grade and performance status as having prognostic relevance for PFS and OS, as was the case with baseline hemoglobin levels. There was no preponderance for any histological subgroup in the long-term responder or survivor patient groups. These findings, consistent with results from other case series summarized in Table 1, indicate that a proportion of patients across many different STS subtypes obtain a significant benefit from pazopanib.

### Radiological biomarkers

Data from the PALETTE study indicate that the PFS gain seen with pazopanib is associated with a minor increase in the rate of radiological objective response compared to placebo (6% vs. 0%) but a much larger increase in stable disease rates (67% vs. 38%)^6^. This finding implies that pazopanib benefit was predominantly associated with disease stabilization rather than significant tumor shrinkage. However, given that over one-third of patients who received placebo also exhibited stable disease as the best response, the difficulty in discerning intrinsically indolent but pazopanib-resistant disease versus true pazopanib-related tumor stabilization presents a clinical challenge when attempting to appraise early signs of benefit to individual patients. The use of imaging modalities that provide information on changes in tumor physiology beyond a dimensional response shows promise for assessing pazopanib effect and represents a potential avenue of delineating the heterogeneity of tumor response within the RECIST category of stable disease. Since responding sarcomas can undergo a decrease in tumor blood supply and consequent cystic degeneration, potentially with an associated increase in tumor dimension due to resulting inflammation, the measurement of changes in tumor vascularity as a proxy for antitumour effect has been pursued. The initial pazopanib phase I trial used DCE-MRI to measure changes in tumor vascularity in a subset of pazopanib-treated patients. Further investigation of this functional MRI modality is required to assess the adequacy of detected vascular alterations as surrogates for survival endpoints. Computerized tomography (CT)-based criteria that account for a reduction in tumor density that reflects tumor cell death have been developed by Choi et al.^39^. These criteria have been demonstrated to be more sensitive for TKI benefit than RECIST criteria in GIST and have shown promise for other STS subtypes in terms of specificity in predicting pathological response and favorable outcome in patients treated with cytotoxic chemotherapy^40–42^. Durable disease control with pazopanib has been reported in patients with SFT who had SD as the best response by RECIST but PR by Choi criteria^43,44^. Following these observations, a prospective, single arm phase II trial of pazopanib in advanced SFT performed at centers in Italy, France, and Spain assessed the overall response by Choi criteria as its primary endpoint^45^. Objective responses by Choi criteria were seen in 18/35 (51%) of treated patients, compared to 2/35 (6%) by RECIST. While Choi criteria and RECIST-based nonprogressing patients exhibited similarly favorable OS, Choi criteria were better than RECIST for identifying patients with progressive disease (PrD) and poor OS (median OS of 4.5 vs. 6.5 months in the Choi PrD and RECIST PrD groups, respectively), and the median OS in patients identified as PrD by RECIST but PR by Choi was 24 months. These data suggest that Choi criteria may better discriminate between patients with or without beneficial responses to pazopanib in the treatment of SFT. Further information on the utility of Choi criteria, as well as functional MRI and fluorodeoxyglucose positron emission tomography (FDG-PET), in predicting pazopanib benefit will be provided by a now-completed window-of-opportunity study of preoperative treatment in localized STS (NCT01543802)^46^. The potential utility of FDG-PET-CT in this scenario is also informed by small studies that have demonstrated an association with either a decreased maximum standardized uptake value (SUVmax) during therapy and durable disease control or the absence of a SUVmax response and lack of clinical benefit^47,48^. Given that Choi criteria, PET-CT, and functional MRI lack validation in this setting and face issues of interobserver reproducibility, further development of these imaging approaches as a tool for accurate early detection of pazopanib benefit is required. However, these modalities remain promising avenues for distinguishing between responding patients who are likely to benefit from ongoing therapy and those without a response who would be better served by a change of therapy.

### Markers of drug exposure

Numerous PK factors can influence systemic drug exposure, including oral absorption and metabolism, drug–drug interactions, food–drug interactions and patient characteristics such as age, gender, and bodyweight, which may be related to variations in the volume of distribution. As such, high interpatient variability of plasma exposure to oral anticancer drugs, including pazopanib, may affect drug efficacy and treatment outcome. Retrospective analyses of clinical trial data on the use of pazopanib in the treatment of mRCC and advanced GIST have reported associations between higher trough levels of plasma pazopanib concentration and longer PFS^49,50^, giving rise to the possibility that variability in drug exposure levels may, at least in part, explain the variability in outcome in pazopanib treatment of advanced STS. A prospective cohort study of patients with mRCC or advanced STS/GIST treated with TKIs including pazopanib demonstrated that dose optimization in response to suboptimal trough drug levels was successful in attaining subsequent adequate trough levels, indicating that dose monitoring and adjustment could represent a path to improved clinical effectiveness of these drugs^51^.

Hypertension is a frequently occurring on-target side effect of pazopanib and correlates with the PK degree of drug exposure. As such, the use of hypertension as a biomarker for adequate drug dosing and/or treatment effect has been pursued. However, as has been seen with the use of pazopanib in mRCC, the combined analysis of prospective patient data from the EORTC phase II and phase III trials has indicated that the development of hypertension during pazopanib therapy is not associated with improved PFS or OS^52,53^. A later analysis of other on-target toxicities within the same cohort found no association between the incidence of drug-induced proteinuria, hypothyroidism, or cardiotoxicity with survival^54^. These findings are consistent with data from a single center 26-patient case series that found no significant association between suboptimal trough levels of pazopanib and worse PFS following treatment, indicating that drug exposure alone is unable to account for variation in pazopanib effect^55^.

In light of the established PK effect of gastric acid suppression (GAS) on many anticancer TKIs, including pazopanib, Mir et al. recently reported a further analysis of the EORTC trial cohorts that investigated a possible association between the concomitant use of proton pump inhibitors or histamine H2-receptor antagonists and pazopanib efficacy^56^. They found that patients who were coadministered GAS for >80% of the duration of their pazopanib therapy had significantly shorter PFS and OS compared to patients who received pazopanib and took either no or less frequent concomitant GAS. These associations were found to be independent of other prognostic factors by multivariate Cox analyses. No association between GAS and survival outcome was found in patients who received placebo, suggesting a causative interaction between GAS use and reduced benefit from pazopanib. However, such conclusions should be tempered by the limitations of the post hoc, retrospective design of this study, wherein correlative PK data were not available, no formal testing for an interaction between GAS exposure and pazopanib benefit compared to placebo was performed, and no association between GAS use and on-target pazopanib toxic effects was observed.

### Baseline biological markers

Due to the inhibitory activity of pazopanib against a number of molecular mediators of tumor angiogenesis and the relative ease of using multiplexed antibody-based assays to assess for protein levels in blood, biomarker research has so far largely focused on investigating circulating angiogenic factors (CAFs). This is especially true in mRCC, a disease where dysregulated angiogenic pathways are known to contribute significantly to tumor development, and for which pazopanib and other TKIs that target angiogenic mediators are established standards of care. These studies have identified a varying repertoire of CAFs or cytokines whose baseline level or change in response to therapy have been associated with differential outcomes following TKI therapy^57^. However, the retrospective nature of many of these studies and the lack of interaction between biomarker and treatment effects in prospective studies have limited the extent to which such investigations inform the search for predictive, rather than prognostic, biomarkers for pazopanib. An exception to this situation was seen in the analysis of prospectively collected pretreatment blood samples taken from patients with mRCC treated in either a noncomparative phase II or randomized phase III study of pazopanib^58^. After initially screening for the association of 17 CAFS with outcome in the phase II cohort, seven candidates were then taken forward for validation in the phase III cohort. Among these, interleukin-8 (IL-8) and osteopontin were validated as negative prognostic, but not predictive, biomarkers for PFS. However, the effect of interleukin-6 (IL-6) levels on post pazopanib outcome was shown to significantly interact with pazopanib exposure, indicating that while patients with an increased baseline IL-6 had a worse prognosis than those with low IL-6, the former was the group in which pazopanib treatment delivered the most benefit. Despite this high-level evidence for baseline circulating IL-6 levels for pazopanib in mRCC, this biomarker has not impacted routine practice so far, for reasons that include the limited analytical replicability of IL-6 assays and the lack of validation in an independent prospective cohort. Other studies have investigated tumor-based pazopanib biomarkers, including retrospective assessment of gene expression-based mRCC molecular subgroups, hypoxia-inducible factor (HIF) levels or von Hippel–Lindau tumor suppressor gene (*VHL)* mutational status in tissue series. These studies have indicated prognostic associations of the investigated markers, but due to their retrospective design and lack of validation, their predictive utility remain unproven^59,60^.

Several studies have reported baseline biomarkers with putative predictive associations with pazopanib efficacy in advanced STS patients. Sleijfer et al. examined the serum levels of cytokine and angiogenic factors in a cohort of 85 patients from the EORTC phase II trial and demonstrated that increased baseline plasma levels of proangiogenic hepatocyte growth factor (HGF) and basic nerve growth factor (bNGF) were associated with worse PFS in pazopanib-treated patients^61^. These findings are consistent with the role of pazopanib as an antiangiogenic agent and an association of various CAFs with poor post pazopanib survival, but the impact of the study is limited by high reported false discovery rates combined with lack of validation in independent cohorts.

The ratio of neutrophils to lymphocytes (NLR) in the circulation of patients can serve as an easy-to-measure marker of systemic inflammatory state in cancer patients. High NLR has been shown to be a negative prognostic marker in multiple solid tumor types^62^. Blood samples collected at pretreatment baseline and after 50 days of therapy were used to assess the association between NLR and pazopanib outcome in 333 patients treated with pazopanib within the EORTC phase II and III studies^63^. While elevated NLR at baseline was a poor prognostic marker regardless of treatment with pazopanib or placebo, no observed pattern of change in NLR between baseline and Day 50 (stable, >40% increase or decrease) was seen to have any association with PFS or OS. In contrast, in a smaller study of 25 patients treated with pazopanib in several Japanese centers, a decrease in NLR from baseline to Week 4 of therapy showed a highly significant association with improved PFS, while baseline NLR had no prognostic association^64^. This study used a higher cut-off to define high and low NLR patients (based on the cohort median NLR value) and did not provide a definition as to what constituted a significant change in NLR during therapy, potentially contributing to the discrepancy with the EORTC cohort analysis. Regardless, beyond the consistently observed prognostic association of NLR in STS and other cancers, its role as a potential predictive biomarker for pazopanib appears to have little promise.

In a study by Koehler et al. next-generation sequencing (NGS) was used to sequence 405 cancer-related genes in pretreatment tumor samples from a retrospective cohort of 19 patients with advanced STS treated with antiangiogenic agents (18 pazopanib and 1 sunitinib)^65^. *TP53* and *RB1* were the only two genes found to be altered in >20% (in 10 and 6 patients, respectively), with all detected mutations of *TP53* predicted to confer loss-of-function (missense mutation of DNA binding and/or tetramerization domain, or homozygous deletion). While *RB1* mutational status had no association with post-pazopanib outcome, patients with *TP53*-mutated tumors were shown to have significantly longer PFS than those with *TP53* wild-type tumors. These data represent the only currently reported tumor-based candidate biomarker for pazopanib in advanced STS and have yet to be validated. The biological basis of any association between *TP53* function and pazopanib response remains to be determined.

### Preclinical evidence of markers of pazopanib sensitivity and resistance

Given that pazopanib selectively inhibits several growth-promoting RTKs, the expression levels of these targets in tumor cells are also attractive candidates for evaluation as predictive biomarkers. While translational studies of tumor-based expression of RTKs in pazopanib-treated STS are lacking, several preclinical studies have assessed the association between RTK expression and pazopanib effect. In one such study, screening of 14 cell lines representing eight different sarcoma subtypes identified that only the two malignant rhabdoid tumor (MRT) cell lines displayed pazopanib sensitivity^66^. These cell lines were shown to express phosphorylated PDGFRA and FGFR1, both kinase targets of pazopanib. Dual pharmacological inhibition or genetic silencing using RNA interference (RNAi) of these targets led to a synergistic increase in tumor cell apoptosis. Acquired pazopanib resistance was then derived in these cells through culture in the presence of an escalating drug dose. Comparison of molecular profiles between parental and resistant cell lines identified significant downregulation of PDGFRA but maintenance of FGFR1 expression and activation. These cells were no longer dependent upon PDGFRA signaling but remained sensitive to FGFR1 inhibition, indicating that loss of PDGFRA could serve as a marker of acquired pazopanib resistance that could potentially be therapeutically targeted with inhibitors of FGFR1. In a follow-up study, further comparative mass spectrometry-based phosphoproteomic characterization of these paired pazopanib-sensitive and pazopanib-resistant cells identified increased activity in cytoskeletal regulatory pathways and downregulation of histone deacetylase activity in pazopanib-resistant cells^67^. Elsewhere, immunoblot and antibody array-based comparative proteomic profiling was performed on four different synovial cell lines, three of which were sensitive to pazopanib, with the other showing primary pazopanib resistance^68^. Increased expression of PDGFRB and phospho-activation of tyrosine kinases, including FGFR3 and VEGFR1, were shown to be unique to the resistant cell line, while RNAi-mediated silencing of PDGFRB, MET, and protein tyrosine kinase 2 beta (PTK2B) reduced viability of the pazopanib-resistant cells. Collectively, these studies indicate that the relative pazopanib sensitivity of sarcoma cells is reflected and possibly determined by differential expression and phosphorylation of RTKs and downstream pathway signaling proteins. While translational correlates of these preclinical findings are lacking, there is an indication that investigating the expression levels of molecular targets of pazopanib within tumor tissue may yet identify biomarkers for drug effect.

## Conclusions and future directions

The successful clinical development of pazopanib as a treatment for advanced STS has addressed the longstanding and ongoing need for effective novel agents for these rare diseases. However, reported clinical trial data indicate that only a minority of patients within the indicated STS population will receive benefit from treatment and that the duration of benefit may in many cases be short. Reported data from subgroup analyses of prospective pazopanib studies have so far failed to identify baseline clinicopathological characteristics that enrich for pazopanib benefit. Moreover, the small number of translational studies that have investigated circulating or tumor-based biomarkers have yet to provide reproducible and validated candidate biomarkers. A growing volume of biomolecular profiling data indicate the existence of biological intrinsic subgroups within individual STS histotypes. In addition, biological traits such as increased chromosomal instability can be demonstrated in a proportion of tumors across multiple STS subtypes and are associated with shared clinical phenotypes^69–71^. Assessment for differential treatment outcomes between such biologically classified STS subgroups within pazopanib-treated cohorts represents a worthwhile avenue for biomarker research.

The further development and validation of putative imaging surrogate markers for survival would provide better discriminatory trial endpoints and assist in the early detection of clinical effects. At present, it is unclear by what precise mechanism or mechanisms pazopanib exerts its anticancer effect in STS and whether this effect varies between and within different STS subtypes.

Furthermore, even in patients showing an unequivocal initial benefit from treatment, the development of pazopanib resistance is ubiquitous. A greater understanding of the mechanisms of drug effect and primary and secondary drug resistance is required to inform patient selection and the development of novel combinatorial regimens in which pazopanib might be paired with other small molecule inhibitors, cytotoxic drugs, or potential immunotherapeutic approaches. A greater degree of preclinical and translational research is required to reveal the therapeutic and resistance mechanisms of pazopanib in disease-specific contexts.
